# A Developmental Psychobiologist’s Commentary on the Future of Affective Science

**DOI:** 10.1007/s42761-024-00273-x

**Published:** 2024-09-19

**Authors:** George F. Michel

**Affiliations:** Psychology Department, University of North Carolina, P.O. Box 26170, Greensboro, NC 27402-6170 USA

**Keywords:** Development, Dynamic systems, Developmental psychobiology, Emotions

## Abstract

A developmental psychobiologist commented on the role of dynamic systems theory in the future of *affective science* and proposed an alternative paradigm.

Shiota et al. (2023) present the rich theoretical and empirical history of affective science but reveal that its core focus is poorly defined: “we have yet to find a simple answer to the question of how and why different emotions and aspects of emotion are connected” (p. 432). Despite controversy about what emotions, feelings, moods, etc., are, there is agreement that Affective Science addresses some complex set of psychological attributes that motivate and enable people to function effectively within a social setting. Since affective science is pursued using multiple methods involving physiological and behavioral measures as well as various forms of self-report, Shiota et al. reasonably call for the establishment of a consensus about the use of appropriate measures, procedures, statistical techniques, etc.

Wood and Coan (2023) argue that dynamic systems theory (DST) could bring theoretical and methodological coherence to affective science. In persuasive detail, they demonstrate how constructs from dynamic systems theory (with the addition of the process of allostasis) can define emotions, account for the transitions among their expression, and avoid the nature-nurture confusion about development. They show how DST permits emotions to appear to be discrete and adaptive despite emerging during development from the affordances of the environment and the physiology and biomechanics of the body. As a developmental psychobiologist, I agree with their developmental emphasis but foresee several problems with adopting DST as a model for affective science.

One problem is historical. Since the 1980s (cf., Oyama, 1985), developmental scientists have proposed using DST as a conceptual scheme for characterizing the development of behavioral phenotypes. In 1985, Fogel (1985; Fogel & Thelen, 1987) employed DST to investigate the development of emotional expression. As do Wood and Coan (2023), Fogel relied on Kelso’s use of DST for characterizing the organization of neuromotor movement (Kelso & Tuller, 1984; Thelen et al., 1987). The organization of complex actions is largely determined by ensembles of neuromotor “coordinative motor structures” (Kugler et al., 1980). Coordinative structures occur because the movement of one set of muscles may recruit the involvement of other muscles depending upon the degree of differentiation among the neural systems activating those muscles. These structures are constrained to cooperate or compensate for each other depending upon the context of their elicitation. These coordinative structures are not programmed by the nervous system but rather emerge from the dynamic of the action itself (Michel, 1991).

Linda Camras and I tested the concept of coordinative structure as a mechanism in the formation of facial expressions of emotion using 5- to 7-month-old infants (Camras et al., 1996; Michel et al., 1992). We found that expressions of “surprise” and “interest” for infants at this age reflected “coordinative structures” involving activation of muscles controlling the neck recruiting muscles of the eyebrows and mouth. Our study indicated that the facial actions of mouth opening, brow raising, and upper eyelid lifting constitute a coordinated motor ensemble with head movements that sometimes operate as an attentional or appetitive response rather than as an expression of emotion.

Adults readily interpret the “facial expressions” derived from these head and gaze coordinative movements as expressions of emotion (Camras et al., 1993). Adults typically provide relatively consistent feedback (vocalizations, facial movements, and manual manipulation of the infant) that can serve as a means for shaping these actions into reliable facial expressions for communicating the individual’s state and desires. Since specific facial actions can be associated with the movement of muscles controlling the head, eyes, limbs, or trunk and are available for recruitment in a variety of circumstances, we concluded that unique and exclusive ties between facial expressions and emotions may never be formed.

In designing our study, we were fortunate to have precise definitions of facial expressions and a fairly rich account of the infant’s neuroanatomical and neurophysiological status. This account indicated that there was considerable axonal arborization of the neurons in the brainstem that activate muscles in the face and neck area during early infancy. Thus, we expected to find evidence of recruitment of facial muscle activation as a consequence of increased activation of neck muscles. This generally poor differentiation of muscle activation has long been observed in the behavior of neonates and infants (Peiper, 1963) and is often called “overflow” or “associated” movements. This lack of differentiation continues well into adolescence, and its rate of disappearance is used as a measure of neurological development (Connolly & Stratton, 1968; Wolff et al., 1983). Even among adults, associated movements can emerge in situations of high neural arousal (e.g., lifting a heavy weight often results in over-tightening of the grip and facial grimacing) or in situations of highly focused attention (e.g., raising the head and eyelids when looking in a mirror to examine the eye or when applying an eye liner recruits eyebrow raises and mouth opening).

In DST, altering a system’s state requires fairly precise knowledge of what constitutes its components and the character of their current dynamic relations (i.e., degree of coherence). To be useful, the set of component elements in any dynamic system must be accurately determined. A roulette wheel seems to operate with complete randomness with the slot in which the ball settles to be unpredictable. However, roulette dynamics represent a deterministic system. Its unpredictability stems from a multitude of influences and its sensitivity to its initial conditions: ball position at dropping, force of the drop, rotation speed of the wheel, position of wheel at initial ball contact, friction “stickiness” of the air and the slots in the wheel, vibration of air around the wheel from customer actions and HVAC fans, etc. Variations in these initial conditions lead to variations in outcomes that fit various stochastic models and create the appearance of random events.

Many dynamical systems exhibit nonlinearity between input (cause) and output (effect) — their behavior becomes more complex and less predictable. Also, although input to the system may increase steadily, a dynamic system can exhibit distinct “phase transition” among its characteristics, producing emergent properties. Phase transitions and the sensitivity of nonlinear systems to minor changes (and their resistance to major changes) account for the non-predictability of the outcomes to input (perturbations).

When engineers create devices that involve dynamic systems, they must make the devices operate with a certain frequency of success. That success can only be determined by using tested mathematical algorithms, previous experimental information, concurrent testing, and the estimation that unusual conditions will occur with very low frequency (it is unlikely that a shuttle will be launched during an unusual freeze in Florida that might crystalize the O-rings securing a rocket booster causing it to ignite). When Kelso used coordinative structures to characterize neuromotor control of the hands, he used common engineering algorithms to make predictions about the overall pattern of movement and its phase transitions as a consequence of increased activation. Then, he tested those predictions with empirical manipulations.

Wood and Coan suggested that emotional states emerge from the interaction of component elements that include cognition and contextual experience. Interactions among the components enhance and constrain specific aspects of each other and thereby give rise to an emergent affective state (emotion, mood, etc.). However, defining affect as an emergent state whose components include cognition and experience creates a challenge. As an emergent phenomenon, affect cannot affect the states of its constituent components (cognition and experience). If affect is to affect cognition and experience, then it must be a component of a broader system that includes cognition and experience as components. At present, too many aspects of affect, cognition, and contextual experience are poorly defined and measured. Without the ability to measure these constructs and test predictions derived from DST, DST becomes just another metaphor for characterizing emotions.

Also, affective scientists need to know the coherence of the components of a system if they are to have some ability to predict and control the system’s emergent properties. Dynamic systems are internally coherent; i.e., changes in their states are primarily determined by forces internal to the system (the dynamic relations among components) rather than just by factors external to the system. This aspect of DST would be a major problem for devising strategies and tactics needed for self- and other-regulation of emotion/mood/affect (Petrova & Gross, 2023). Internal coherence among components refers to the relation among the dynamics of each component (its frequency of oscillation and state of damping or amplification). Do the components have the same phase relation or are they displaced? Do they have the same frequency or are they harmonic or discordant? Are the components’ amplitudes differentially damped or amplified?

Components will vary in one or more of their features making them discordant and more susceptible to external perturbations. Perturbing events external to the system can produce change, but the nonlinearity between the degree of perturbation and the likelihood of change means that the perturbation’s influence on affective states is predictable only from the knowledge of the degree of coherence of the components of the system responsible for these states. Of course, highly traumatic events such as major catastrophes can profoundly affect an individual regardless of the state of coherence of the system. However, most self- and other-regulatory strategies and tactics are seldom at that level of perturbation (for an account of successful amelioration of the traumatic developmental effects of war-induced orphanhood, see Wolff, 2023).

Perhaps, we do not need DST in order to effectively understand the development of affect. Consider that for everyday life, causality usually reflects metaphors of human agency — manipulation and control. We look for aspects of nature that provide a “handle” for our manipulation and control. To avoid contracting the Covid virus, we socially isolate, wear masks in public, wash hands frequently, and reduce human contact. These are things that we can control and manipulate. We can experimentally test the “effects” of such control on the likelihood of contracting Covid. Thus, unlike DST, cause for us applies to events in nature that make some difference to some effect we are seeking and provide us with a sense of prediction and control.

The nature-nurture controversy has provided a set of research paradigms that produce a sense of prediction and control. If a psychological attribute appears early in development, we consider it to be the effect of processes that are independent of the individual’s experience. If we do not notice any instance of training, imitation, or practice before manifestation of an attribute, then it is likely the effect of processes independent of experience. If the behavior is rewarded, modeled, or encouraged by the culture/family/society of the individual, we can consider its manifestation to depend upon experience. Applying the meme of nature-nurture interaction, we can side-step how such interaction occurs. These and a number of other causal “handles” allow Affective Science to suffer from a nature-nurture tension when seeking to understand the development of affective expressions, states, or concepts.

However, developmental psychobiology (DPB) provides a set of research paradigms that enable prediction and control of the development of species-typical behaviors while avoiding the nature-nurture dead-end (Michel & Moore, 1995; Michel et al., 2015). For DPB scientists, development always derives from the *co-action* of physiological, biomechanical, and environmental processes operating during earlier phases of development. Although it is not possible for one team to thoroughly investigate the specifics of each of these co-acting processes, the investigation of each and their coaction is always encouraged.

The nature-nurture conundrum has been maintained by an intentional stance (or “development to” approach) in research that considers development as proceeding to attributes (Dennett, 1987). In contrast, DPB adopts a historical stance or “development from” approach, (Michel & Tyler, 2007; Moore, 2003): Development builds upon earlier phases whose attributes are identified independent of any particular foreseen end-state. From the intentional stance, facial expressions of emotion are sought in earlier developmental phases (e.g., in utero) as evidence of their adaptive function and control by “nature.” From the historical stance, early forms of facial activity (”expressions”) are a feature of the early behavioral repertoire. These may or may not be found to develop later into what we consider to be emotional expressions. If they do, then they are one of the bases upon which expressions of emotion are developed. Thus, DPB encourages multiple methods of investigation (e.g., measures of physiological, biomechanical, and environmental processes, including social feedback) that can reveal the developmental causes/bases of affect without focusing on their status within the nature-nurture controversy.

## Additional Information

### Data Availability

No datasets were generated or analysed during the current study.
